# Rhinosporidiosis: Intraoperative Cytological Diagnosis in an Unsuspected Lesion

**DOI:** 10.1155/2012/101832

**Published:** 2012-10-11

**Authors:** Shruti Bhargava, Mohnish Grover, Veena Maheshwari

**Affiliations:** ^1^SMS Medical College, Jaipur, India; ^2^JNMCH, AMU, Aligarh, India

## Abstract

Rhinosporidiosis is a disease endemic to South India, Sri Lanka and some areas of the African continent. The nasal lesions can sometimes be confused with nasopharyngeal malignancy. We report here a clinically unsuspected case of rhinosporidiosis, diagnosed correctly by intraoperative FNAC, and later confirmed by histopathological examination.

## 1. Introduction

Rhinosporidiosis is a rare chronic granulomatous disease of mucocutaneous tissue, endemic in South India, Sri Lanka, and some areas of the African continent [1]. The etiological agent *Rhinosporidium seeberi*, in recent studies has been established as an aquatic protistan parasite [2]. It commonly affects the nasal mucosa, conjunctiva and urethra in people of any age and sex, but involvement of other sites has also been reported [3]. In the nasal cavity it manifests as a polypoid mass which can be confused sometimes with malignant lesions, wherein the exact diagnosis is confirmed on histology [2]. However, FNAC is an economical and reliable intraoperative method for diagnosis of such suspected and unsuspected lesions [3]. Surgical excision is the treatment of choice, and recurrence is possible but rare [4]. We report here a case of rhinosporidiosis diagnosed by intraoperative FNAC. 

## 2. Case Report

A 21 years-old-man presented to the ENT outpatient department with obstruction of nose on right side. On examination there was an erythematous, irregular mass, 3 cm in diameter, obstructing the right nasal cavity and extending into the sinuses as well. No abnormality was seen in the contralateral nasal cavity or nasopharynx. 

**Figure 1:**
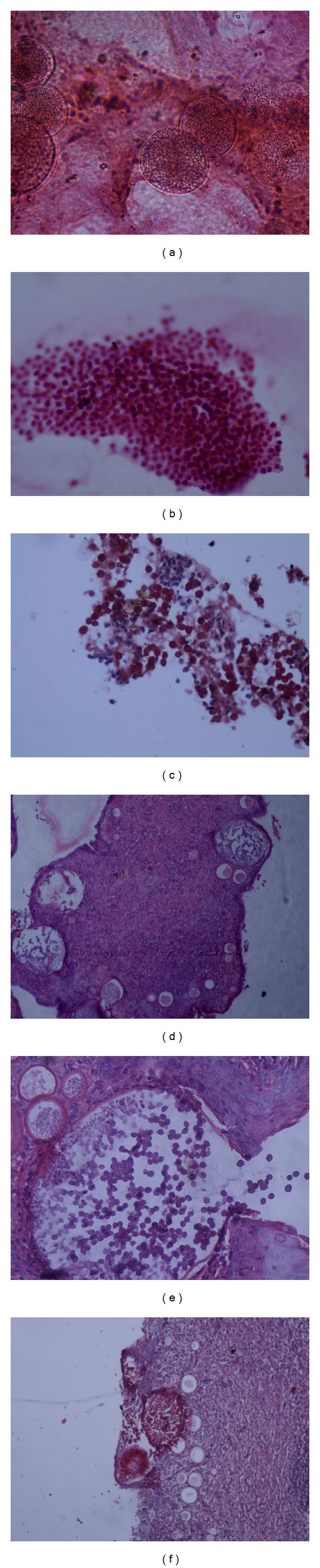
(a) FNA smear showing globular sporangia containing spores (H&E ×400). (b) FNA smear showing isolated spores and inflammatory cells (H&E ×400). (c) PAS positive spores on FNA smear (PAS ×400). (d) Histopathological section showing sporangia and inflammatory cells in a fibrous stroma covered by stratified squamous epithelium (H&E ×100). (e) Ruptured sporangia liberating the spores histopathology section (H&E ×400). (f) PAS positive sporangia and spores on histology (PAS ×100).

Since, preoperatively, the exact nature of mass could not be clinically established with surety, an intraoperative aspirate smear was prepared to rule out malignancy. Rapid Hematoxylin & Eosin (H&E) and Periodic acid Schiff (PAS) stained smears revealed numerous globular sporangia containing spores (Figure 1(a)) along with many free lying spores and inflammatory cells (Figure 1(b)). The spores stained magenta with PAS (Figure 1(c)), a feature used to differentiate them from epithelial cells of nasopharynx (PAS negative). Hence the diagnosis of rhinosporidiosis was established and malignancy was ruled out. This helped the surgeon completely remove the mass endoscopically.

Histopathological examination of the resected mass revealed many globular cysts, each representing a thick-walled sporangium containing numerous daughter spores in a background of fibroblasts and acute and chronic inflammatory cells, covered by flat multistratified squamous epithelium (Figures 1(d) and 1(e)). The endospores and sporangia were PAS positive (Figure 1(f)) and were, respectively, 5–10 *μ*m and 50–1000 *μ*m in size. These findings made easier the distinction of *Rhinosporidium seeberi* from another common nasal mycosis etiological agent, *Coccidioides immitis*. And hence, the intraoperative cytological diagnosis was confirmed.

The patient did not assume any drug therapy and, until today, after one year of followup, during which he, underwent clinical examination twice, remains healthy with no sign of recurrence.

## 3. Discussion

Rhinosporidiosis is a rare disease affecting people of any age and sex [1]. Initially described by Seeber in 1900 in an individual from Argentina, rhinosporidiosis is endemic in India, Sri Lanka, South America, and Africa [2]. 

The great majority of cases are sporadic. The etiological agent *Rhinosporidium seeberi* causes granulomatous inflammation of mucocutaneous sites, presenting most frequently as polypoidal lesions in the nose. Sites like the conjunctiva, trachea, nasopharnyx, skin, and genitourinary tract are less frequently involved [3]. 

The taxonomy of *R. seeberi* was debated in the last decades, since the microorganism is intractable to isolation and microbiological culture [2]. Moreover, its morphological features resemble both fungi and protozoa [2]. Interestingly, the histopathology of some fish and amphibian diseases as well as the morphology of these pathogens closely resembles that of rhinosporidiosis [5]. Recently, it has been classified as the first known human pathogen belonging to class of aquatic protistan parasites [6]. 

The presumed mode of infection from the natural aquatic habitat of *R. seeberi*, is through the traumatized epithelium (“transepithelial infection”) most commonly in nasal sites [2]. There is evidence for hematogenous spread of rhinosporidiosis to anatomically distant sites [2]. 

The nasal lesions may sometimes present clinically as ulcerated growths which could mimic malignant lesions such as sarcomas and carcinomas [2].

The definitive diagnosis of rhinosporidiosis is by histopathology on biopsied or resected tissues, with the identification of the pathogen in its diverse stages, demonstration of sporangia and endospores [2]. The sporangia are large, thick-walled spherical structures (called sporangia) containing smaller “daughter cells” (called “sporangiospores”), seen in a stroma which is either fibromyxomatous or fibrous containing chronic inflammatory cells which include macrophages and lymphocytes, while neutrophils are numerous around free endospores [2]. Each mature sporangium contains an operculum or pore through which the endospores are extruded [2].

However, cytodiagnosis on aspirates from rhinosporidial lumps or on smears of secretions from the surfaces of accessible polyps and fine-needle aspirates from lumps provide, with suitable stains, distinctive diagnostic features [2]. The various developmental stages of sporangia can be readily identified by special fungus stains such as the Gomori methenamine silver, Gridley's, and the periodic acid-Schiff stains, although the identification of the stages can also be made with the routine haematoxylin and eosin stain [2].

On direct examination, the cytological smears show spherules as well-circumscribed, globular structures with several endospores within [7]. The diameter of spherules ranges from 30 to 300 microns. The endospores may be confused with epithelial cells. The PAS stain is used to discriminate between endospores and epithelial cells, in which the residual cytoplasm and large nuclei can sometimes simulate the residual mucoid sporangial material around the endospores and the endospores themselves [2]. The endospores stain markedly magenta while the epithelial cells are PAS-negative [2]. 

*Rhinosporidium seeberi* should be distinguished from another microorganism, *Coccidioides immitis* [2]. This latter has similar mature stages represented by large, thick-walled, spherical structures containing endospores, but the spherules are smaller (diameter of 20–80 *μ*m versus 50–1000 *μ*m) and contain small endospores (diameter of 2–4 *μ*m). Moreover, *Coccidiodes* does not stain with the mucicarmine [2].

The only curative approach is the surgical excision combined with electrocoagulation. There is no demonstrated efficacy in using antifungal and/or antimicrobial drugs. Recurrence, dissemination in anatomical close sites and local secondary bacterial infections are the most frequent complications [4]. 

To conclude, Rhinosporidiosis is a condition which both clinicians and pathologists should keep in mind when managing patients from endemic countries with nasal masses. The cytological appearance of fine needle aspiration smears is distinctive, and a definitive diagnosis of rhinosporidiosis can be made easily and quickly in clinically unsuspected cases.
